# Supplementary material for: Morphological changes in response to environmental stresses in the fungal plant pathogen Zymoseptoria tritici
Source: Sci Rep. 2019 Jul 3;9:9642. doi: 10.1038/s41598-019-45994-3 (PMC6610121; doi:10.1038/s41598-019-45994-3)
Supplement: Supplementary file 1 — Supplementary Information [file 41598_2019_45994_MOESM1_ESM.pdf]

Supplementary Information

Morphological changes in response to environmental stresses in the fungal plant pathogen *Zymoseptoria tritici*

Carolina Sardinha Francisco, Xin Ma, Maria Manuela Zwyssig, Bruce A. McDonald, Javier Palma-Guerrero

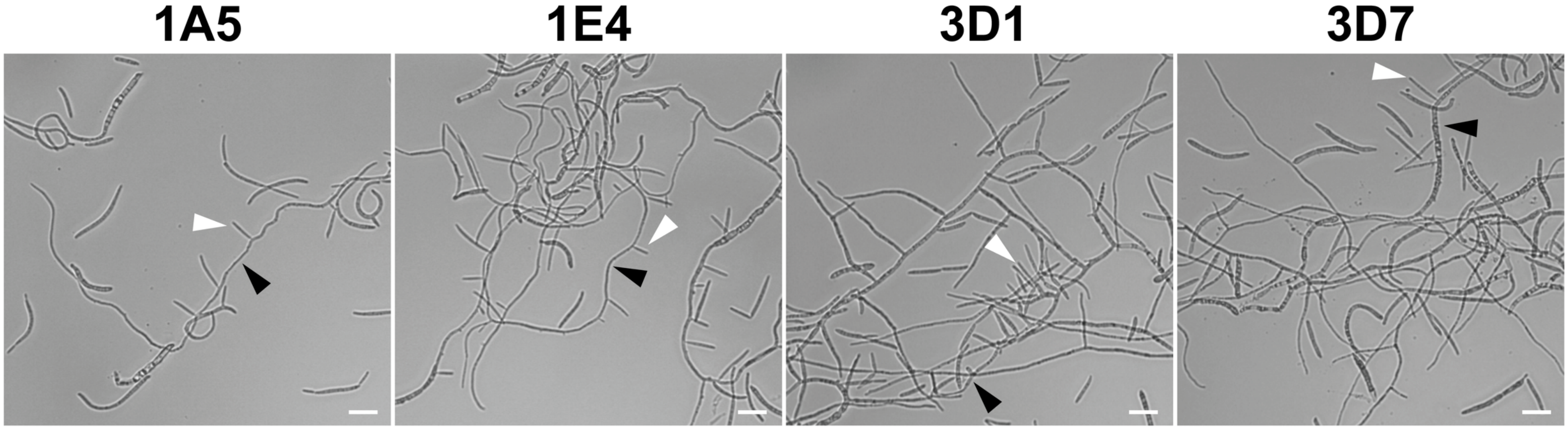

Fig. S1. The morphological transition is bidirectional in *Zymoseptoria tritici*. Regular Minimal Media containing ammonium nitrate and sucrose induced a mixture of morphotypes, such as blastospores, hyphae and budding blastospores (white triangles) from filamentous hyphae (black triangles). Bars represent 30 µm.

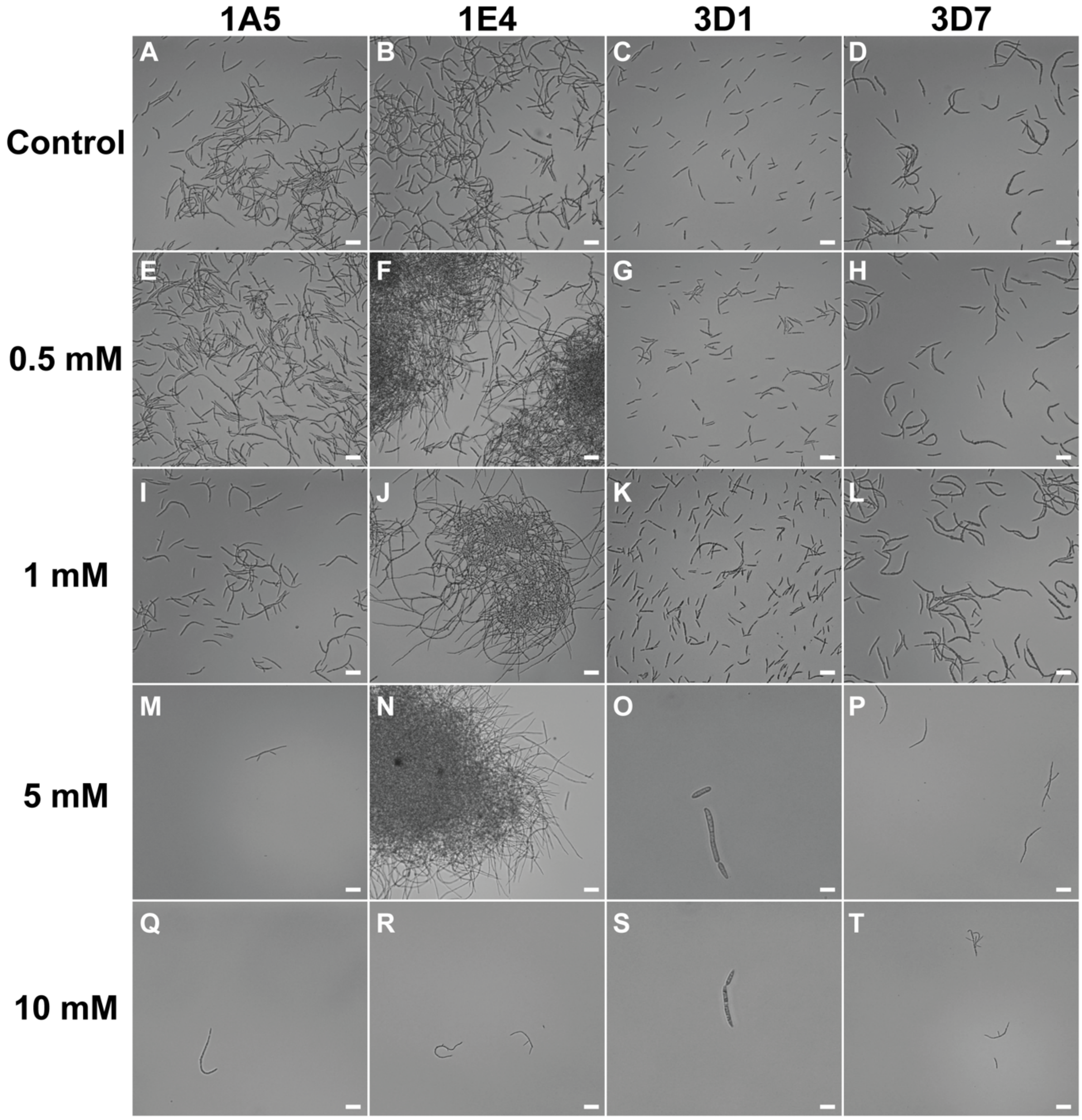

Fig. S2. Effect of exogenous oxidative stress induced by H_2O_2 on the cell morphology of four *Zymoseptoria tritici* strains using a high blastospore inoculum density (10^5 blastospore/mL). A nutrient-rich medium (YSB) containing different concentrations of H_2O_2 was used to incubate the strains under oxidative stress for 72 hours. Blastopores kept on YSB medium without addition of H_2O_2 continue to multiply as budding blastospores via blastosporulation and were used as a control (A, B, C, D - bars represent 50 μm). Despite the increases in H_2O_2 concentration in the medium, no morphological changes were observed for 1A5 (E, I,

M, Q – bars represent 50 μm), 3D1 (G, K – bars represent 50 μm; O and S – bars represent 10 μm), and 3D7 (H, L, P, T - bars represent 50 μm). The 1E4 strain responded morphologically to H_2O_2 concentrations at 0.5 mM, 1 mM, and 5 mM (F, J, N - bars represent 50 μm) undergoing the blastospore-to-hyphae transition. As observed for other strains (Q, S, and T), blastospores of the 1E4 strain remained as undifferentiated structures at 10 mM (R - bars represent 50 μm).

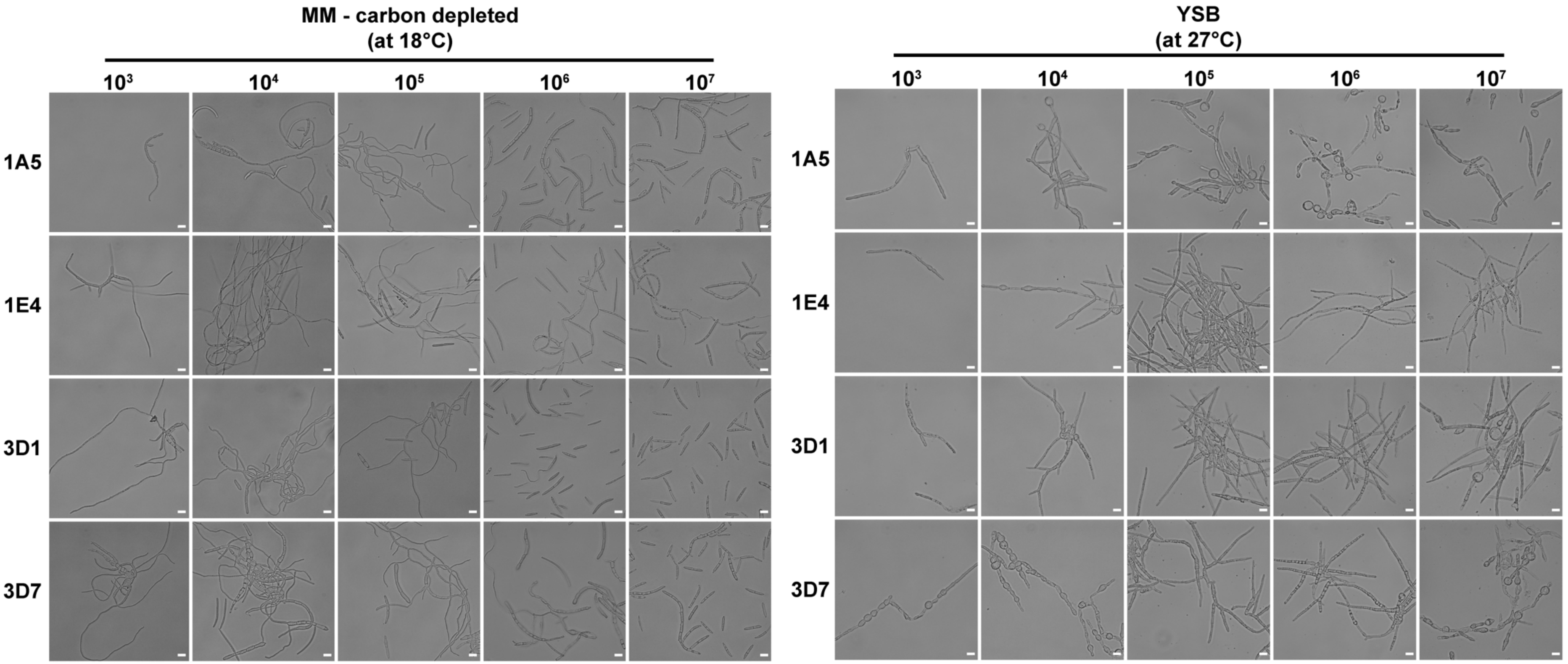

Fig. S3. Cell morphologies of four *Zymoseptoria tritici* strains after 144 hours of incubation on two distinct hyphal induction media and at different initial cell densities. Blastospores incubated in C-depleted MM at 18°C demonstrated a morphological independence of the inoculum size until the concentration of 10^5 blastospores/mL, where they were able to switch to hyphal growth and to form mycelial colonies. At higher initial inoculum concentrations ($\geq 10^6$ blastospores/mL), a drastic reduction of growth form transition and in hyphal length was observed for all tested strains. Blastospores incubated in YSB medium at 27°C showed significant

differences among the strains. At lower initial inoculum concentrations (10^3 and 10^4 blastospores/mL) only pseudohyphae and chlamydospores formed. Intermediate inoculum concentrations (10^5 blastospores/mL and 10^6 blastospores/mL) promoted hyphal growth for the 1E4, 3D1 and 3D7 strains. A ten-fold increase in the initial cell density (10^7 blastospores/mL) favored chlamydospore formation in all strains. 1A5 blastospores were not able to switch to hyphae and undergo transitions to pseudohyphae and chlamydospores for all tested concentrations. Bars represent 10 μm.

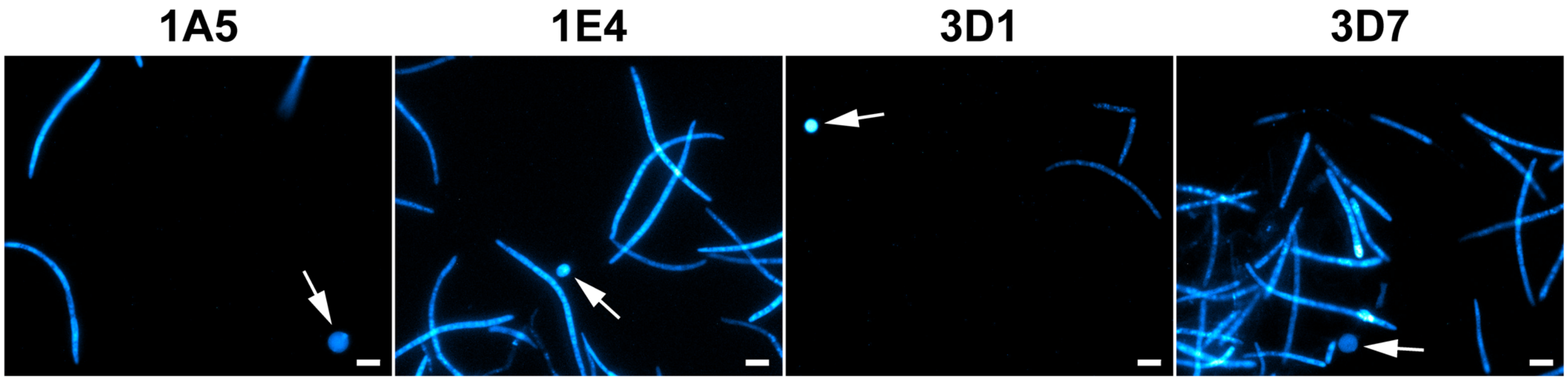

Fig. S4. Chlamydospore cells observed in a spore solution harvested from 20-day- old infected leaves. Plants were previously inoculated with pycnidiospores of four *Z. tritici* strains. Free chlamydospores expressing cytoplasmic GFP were frequently observed mixed with pycnidiospores in the spore solution of all tested strains. Bars represent 30 µm. The proportion of chlamydospores was visibly lower compared to pycnidiospores.

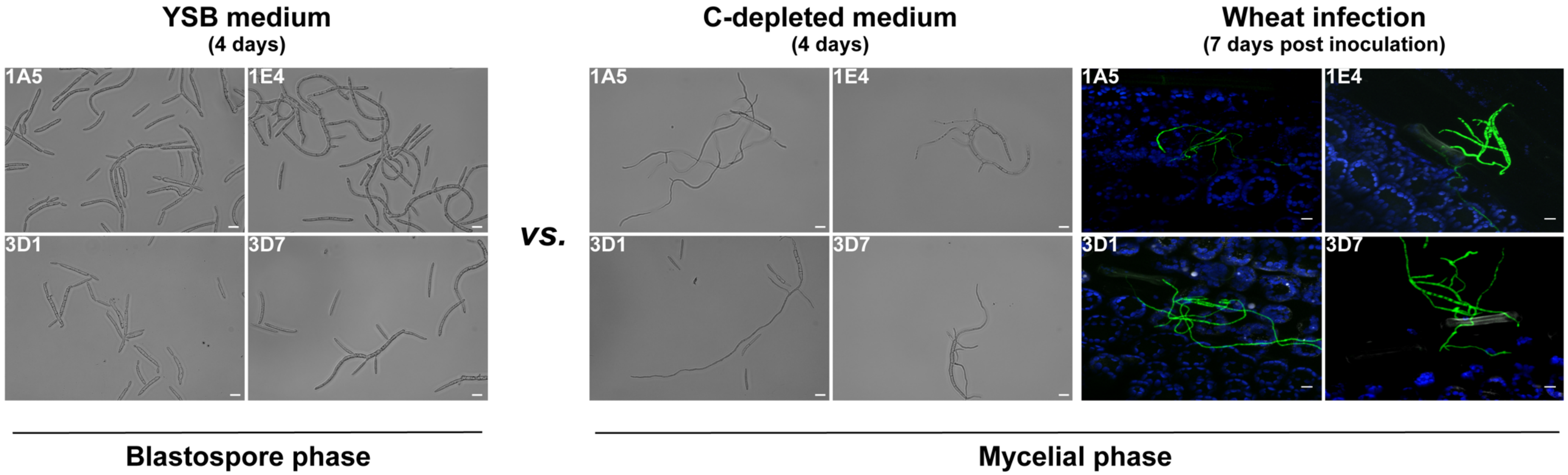

Fig. S5. Morphological forms of four *Zymoseptoria tritici* strains grown in two distinct morphotype-induction conditions used for the RNA-Seq. Nutrient-rich medium (YSB medium) induces only blastosporulation. Nutrient-poor medium (MM C-depleted medium) and the wheat leaf surface induced the blastospore-to-hyphae transition and mycelial growth in all tested strains. Wheat leaves infected with *Z. tritici* strains expressing cytoplasmic GFP (green color) were analyzed using confocal microscopy. The blue color corresponds to chlorophyll A detected from chloroplast autofluorescence. Bars represent 10 μm.

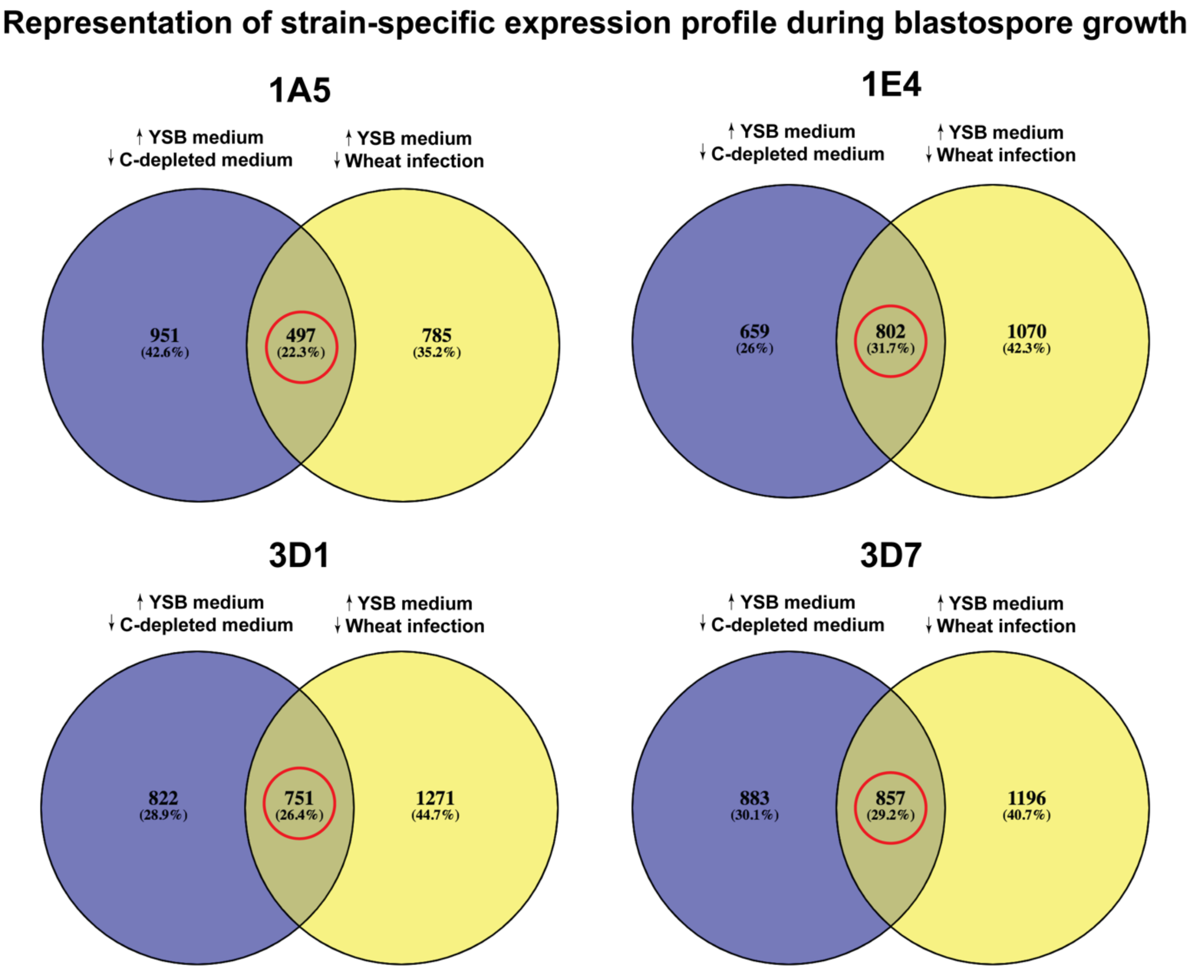

Fig. S6. Representation of strain-specific expression profiles during blastospore growth. Venn diagrams of the overexpressed genes (FDR≤0.01) for each strain grown under blastospore-inducing condition (YSB medium) with respect to hyphae-inducing conditions (C-depleted medium or wheat infection). Arrows pointing up represent the upregulated condition and arrows pointing down represent the downregulated condition. Numbers in red ovals represent the overlapping genes used to identify the “blastospore-related genes”. Numbers in purple or yellow ovals correspond to genes exclusively upregulated in YSB medium compared to C-depleted medium or during wheat infection, respectively.

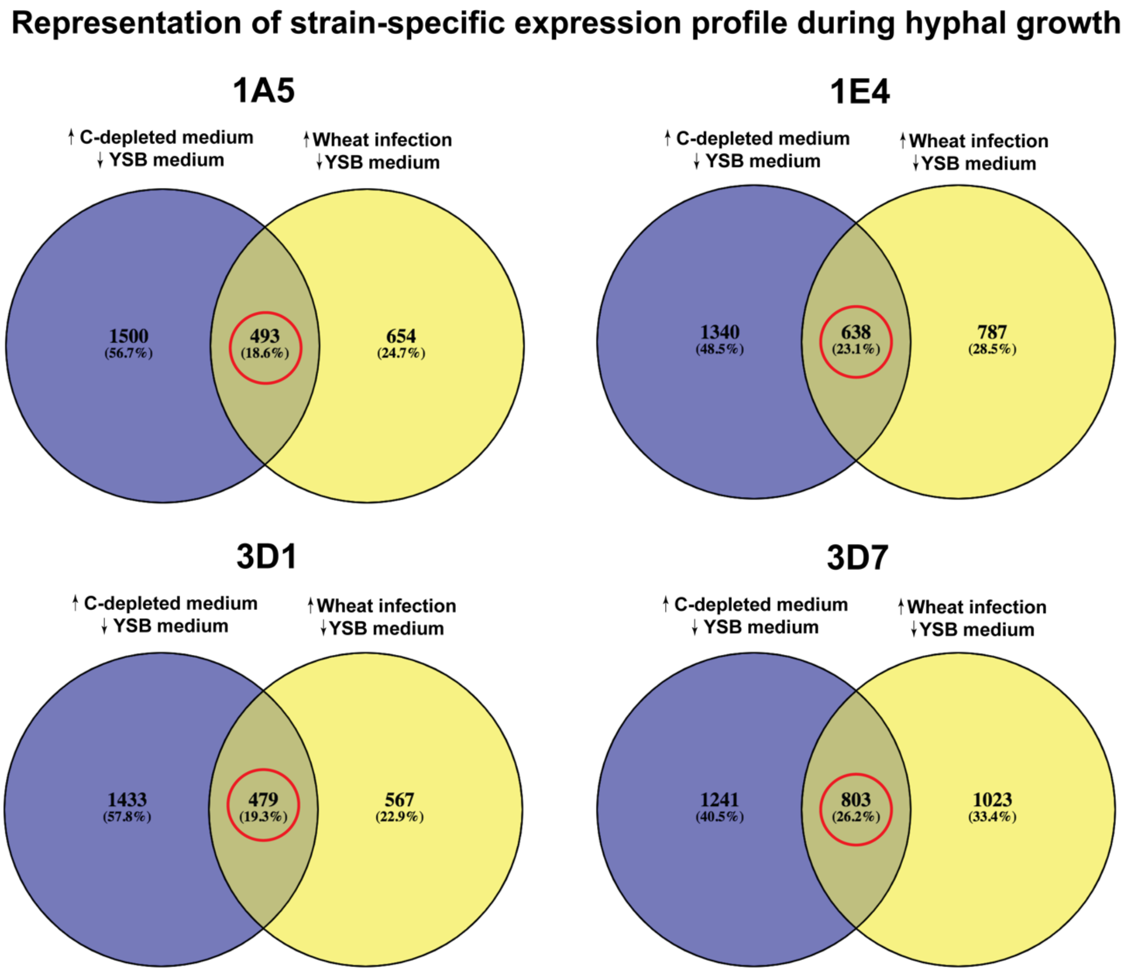

Fig. S7. Representation of strain-specific expression profiles during hyphal growth. Venn diagrams of the overexpressed genes (FDR≤0.01) for each strain grown under hyphae-inducing conditions (C-depleted medium and wheat infection). Arrows pointing up represent the upregulated condition and arrows pointing down represent the downregulated condition. Numbers in red ovals represents the overlapping genes used to identifiy the “mycelial-related genes”. Numbers in purple or yellow ovals correspond to genes exclusively upregulated in C-depleted medium or during wheat infection compared to YSB, respectively.

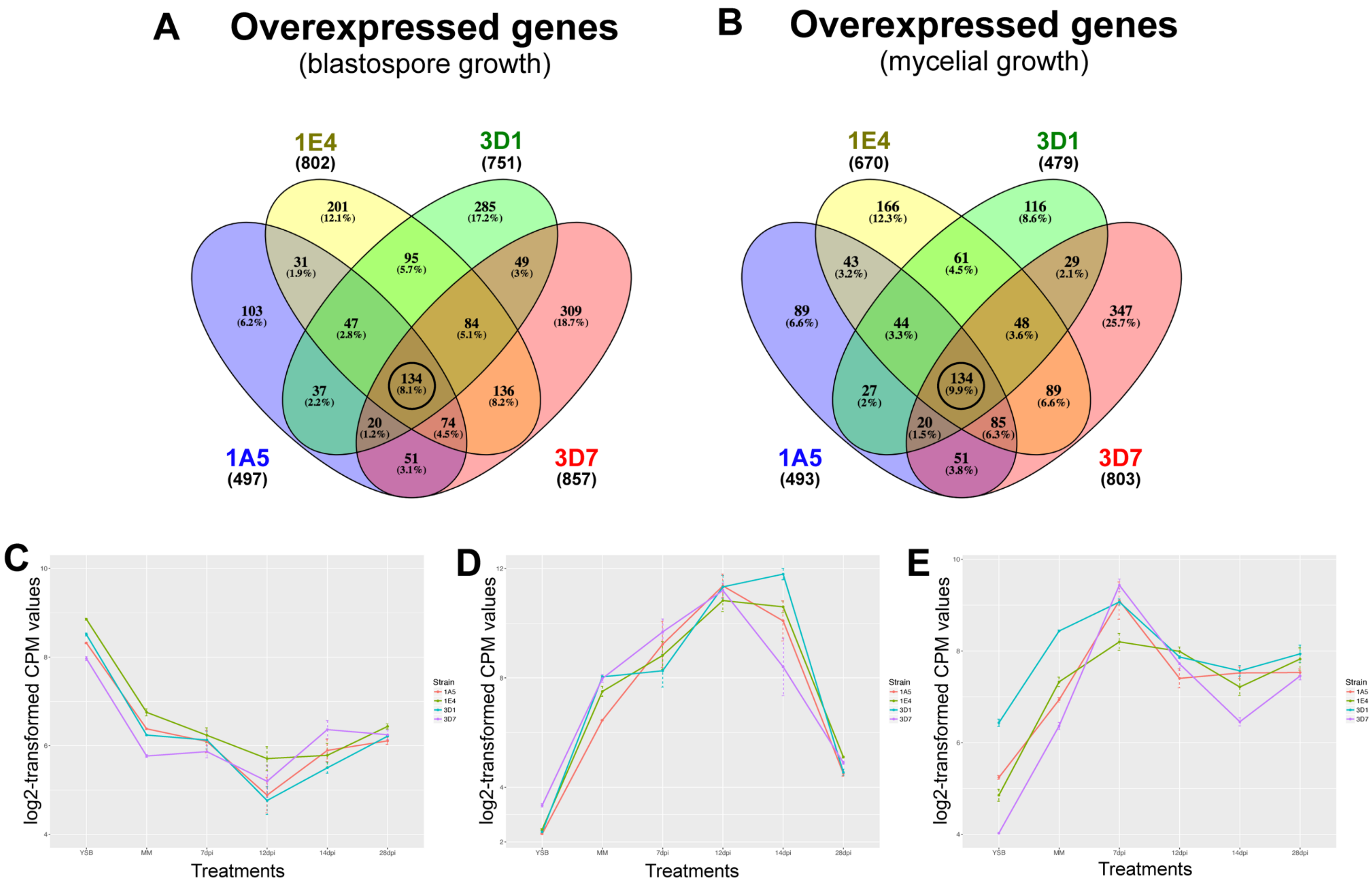
A
Overexpressed genes
(blastospore growth)
1E4
(802)
3D1
(751)
1A5
(497)
3D7
(857)
201 (12.1%)
285 (17.2%)
31 (1.9%)
95 (5.7%)
49 (3%)
103 (6.2%)
47 (2.8%)
84 (5.1%)
309 (18.7%)
134 (8.1%)
37 (2.2%)
136 (8.2%)
20 (1.2%)
74 (4.5%)
51 (3.1%)
B
Overexpressed genes
(mycelial growth)
1E4
(670)
3D1
(479)
1A5
(493)
3D7
(803)
166 (12.3%)
116 (8.6%)
43 (3.2%)
61 (4.5%)
29 (2.1%)
89 (6.6%)
44 (3.3%)
48 (3.6%)
347 (25.7%)
134 (9.9%)
27 (2%)
89 (6.6%)
20 (1.5%)
85 (6.3%)
51 (3.8%)
C
D
E
log2-transformed CPM values
Treatments
YSB
MM
7dpi
12dpi
14dpi
28dpi
Strain
1A5
1E4
3D1
3D7

Fig. S8. Core transcriptome signatures of four *Zymoseptoria tritici* strains from two distinct growth phases. (A-B) Venn diagram of the overexpressed genes (FDR≤0.01) for each strain grown under blastospore and mycelial growth conditions. (C) Graphic representing average expression (log2-transformed counts per million values) of seven genes related to blastosporulation during the blastospore growth phase among strains. (D) Graphic representing average expression (log2-transformed counts per million values) of 39 virulence-related genes during the mycelial growth phase. (E) Graphic representing average expression (log2-transformed counts per million values) of six genes related to cellular morphogenesis during the mycelial growth phase.
